# Albert Bandura's experiments on aggression modeling in children: A psychoanalytic critique

**DOI:** 10.3389/fpsyg.2022.988877

**Published:** 2022-11-25

**Authors:** Evangelia Galanaki, Konstantinos D. Malafantis

**Affiliations:** Department of Primary Education, National and Kapodistrian University of Athens, Athens, Greece

**Keywords:** aggression, modeling, children, Bandura, psychoanalysis, identification with the aggressor, introjection of the aggressor, seduction

## Introduction

In a series of innovative experiments, Bandura (1925–2021), renowned Psychology Professor at Stanford University, USA, and his collaborators (e.g., Bandura and Huston, 1961; Bandura et al., 1961, 1963; Bandura, 1965, 1969) showed that young children exposed to adults' aggression tend to behave aggressively. In these experiments, children observed adults, *in vivo* or *in vitro*, as well as cartoons, behaving aggressively toward a large, inflated doll (clown) named “Bobo doll”, for about 10 min. The findings of these studies are considered to support modeling, observational learning, or learning by imitation and provide evidence for Bandura's social learning theory, which belongs to the behaviorism paradigm. In this paper, we offer a psychoanalytic critique of these experiments with the aim of shedding light on the unconscious processes of children's imitation of aggression. Although Bandura (1986) later formulated the so-called social *cognitive* theory and focused on less observable processes (e.g., self-regulation, self-efficacy, beliefs, expectations), in presenting these early experiments he clearly opposed the existing psychoanalytic interpretations of aggression.

## Key findings of Bandura's experiments on aggression in children

The key findings of Bandura's experiments on aggression in children (Bandura and Huston, 1961; Bandura et al., 1961, 1963; Bandura, 1965, 1969) are summarized below.

1. Observation of an aggressive model is sufficient to elicit aggressive behavior in the young child. The model does not need to be a familiar or nurturant person. Moreover, there is no need to positively reinforce the aggression of either the adult model or the child. Because punishment does not follow the model's aggressive acts, the child receives the message that aggression is acceptable.
2. The virtual world has great power. Children who watch a film showing aggressive people or cartoons tend to imitate this behavior.
3. Imitation is inferred by the fact that children show verbal and/or physical aggressive acts that are very similar to those of the model.
4. Children not only accurately imitate the observed behaviors but also show ingenuity, manifesting different, novice acts of aggression.
5. Children transfer, by means of generalization, aggression into new, different contexts, even when the aggressive model is no longer present (delayed imitation).
6. If the adult model is punished for his/her aggressive behavior, the probability that the child will show aggressive behavior is reduced. In contrast, positive reinforcement or no reinforcement of the model leads to increased aggression on the part of the child (vicarious/indirect learning).
7. After observing the aggressive model, boys tend to exhibit more physical aggression than girls, whereas no gender difference is found for verbal aggression. Independent of gender, children are more likely to imitate a male physically aggressive model. According to gender stereotypes, this form of aggression is more acceptable for men than for women. In contrast, verbal aggression is more likely to be imitated when manifested by a same-sex model.

Taken together, these results imply that children's aggression can be caused—and probably eliminated—by external manipulations. However, are there interpretations other than this omnipotent behavioristic view?

## Psychoanalytic views of children's aggression in Bandura's experiments

In the Bobo doll experiments, after presenting the aggressive model and before placing the child in the room with Bobo doll and other toys with the aim of recording the likelihood of imitation, the experimenters instigated the children's aggression. Specifically, an experimenter led children to another room, where she allowed them to enjoy some attractive toys. After a while, she told them that all toys were hers, that she would no longer let anyone play with them, and that she intended to give them to other children. After experiencing this *frustration*, the children were accompanied to the room where Bobo doll was.

Bandura (Bandura and Huston, 1961; Bandura et al., 1961) stated that he was seeking a more concise and parsimonious theoretical explanation than the one provided by *identification with the aggressor*, that is, the ego defense mechanism described by Anna Freud (1946), and attempted to outline alternative explanations (Bandura, 1969). However, if we look closely at specific aspects and manipulations of these experiments, we may discover that this mechanism may have more explanatory power for what happened in the laboratory than Bandura believed.

At first, it is reasonable to hypothesize that, in the eyes of the children, the experimenters were *omnipotent* adult figures with authority, prestige, and power. The strange laboratory setting may have elicited in children excessive *arousal*, associated with tension and anxiety. This overflow of excitation, that needed to be released, is likely to have resulted from the unprecedented experience, and, more specifically, from the following: separation from parents; presence in an unknown place with strange adults; alternation of unfamiliar rooms and buildings; many overwhelming stimuli, such as physical and verbal aggression exhibited by adults, *in vivo* or *in vitro* (i.e., film), or by cartoons within a colorful frame, full of imaginary stimuli; presence of new and exciting toys; and frustration and anger caused by adults who deliberately disrupted children's pleasurable play activity with the aim of provoking their aggressiveness. All these conditions imply that the experiments were not only about “observation of *cues* produced by the behavior of others” (Bandura et al., 1961; our emphasis). If only “cues” were given to children, then why it was assumed in another paper (Bandura et al., 1963) that vicarious learning had such a “cathartic function”? Indeed, Bandura may have aptly used this expression because *catharsis* implies release of tension caused by *overwhelming vicarious* experience such as in ancient Greek tragedy.

Second, identification with the aggressor is a defense mechanism that is typical of 3- to 6-year-old children—the participants' age in Bandura's experiments. Anna Freud (1946, p. 113) argued that “by impersonating the aggressor, assuming his attributes or imitating his aggression, the child transforms himself from the person threatened into the person who makes the threat”. Children may have unconsciously experienced the aggressiveness of adults (quasi parental figures) toward a familiar playful object as a *threat of punishment*, possibly a *threat of castration by proxy*, for their own oedipal/incestuous and autoerotic/masturbatory phantasies, which usually prevail in this age period—the phallic phase of libidinal development (Freud, 1953). This explanation is further supported by the finding that males were more influential models regarding physical aggression. According to Anna Freud (1946), identification with the aggressor is the preliminary stage of superego formation, during which the aggressive drive is not yet directed against the subject but against the outer world. Projection of guilt, thus, supplements the immature superego and may interpret, at least partly, children's sadomasochistic relation with the doll.

Third, we contend that a *seduction* process of both caretakers and their children had taken place in the university laboratory. With their caretakers' consent, children were brought into an unknown adult place, where they were captivated by adults' passion, namely overt violence against a doll. The violent acts were exhibited in a ritualistic and self-reinforcing manner and in the context of symbolic play. According to Ferenczi (1949), who was not mentioned by Bandura but whose ideas on this issue inspired Anna Freud, when an adult becomes sexually seductive or violent against a child, a *confusion of tongues* between the two emerges, in other words, a confusion between *child tenderness* and *adult passion*. In these experiments, children experienced an indirect attack with a mild traumatic character: certain adults intruded and impinged on the territory of children's “innocent” play, and then coerced them to observe other adults having little control over their own instinctual (aggressive) drives toward an attractive object. Therefore, it was very likely that children reacted not just with imitation but with anxious identification with the adult. This *introjection of the aggressor* resulted in children exhibiting the same violent behavior. They seemed to “subordinate themselves like automata to the will of the aggressor” and “could only react in an autoplastic way by a kind of *mimicry*” (Ferenczi, 1949, p. 228, our emphasis), possibly introjecting the adults' unconscious guilt for their abusive behavior.

It is important to note that, contrary to identification with the aggressor, introjection of the aggressor is initially an automatic, organismic reaction to trauma—a mixture of rage, contempt and omnipotence—and only later becomes a defensive, agentic and purposeful process (Howell, 2014). In these experiments, children seemed to exhibit this automatic, procedural identification and mimicry. It has also been argued (Frankel, 2002) that identification with the aggressor is a universal and very common tactic used by people in *mild* traumatic situations and, generally, on several occasions where they are in a weak position relative to more powerful others. Although benign, this power may become a real threat: “If the adult got out of control and attacked the doll, could she attack me too?” Identification with the aggressor, then, serves an evolutionary function: survival is ensured if individuals conform to what others expect of them.

In the laboratory setting, children confronted what Lacan (1977) has called the *enigma of the adults' desire*: “Why are they behaving this way?”; “What do they want from me?”; “Why are they doing this to me?”. The laboratory setting and the adults' aggression toward the doll can be conceptualized as *enigmatic signifiers*, the Lacanian notion further elaborated by Laplanche (1999). These signifiers were verbal and non-verbal messages, doubly compromised and non-transparent to both sides of the communication because of the existence of the unconscious. The young participants found themselves in an asymmetrical relationship while their developmental abilities to metabolize what adults communicated to them were inadequate. They were somewhat helpless. Aggressive behavior was the way with which children attempted to translate adults' “alien” messages and derive *meaning* from the enigmatic situation.

The ingenuity and novelty—“creative embellishment” as Bandura said when describing the experiment in a short film^1^—which children showed in the aggressive use of toys may be regarded as proof of the playful character of the imitation. Children attempted to *transform passivity into activity*, to acquire mastery of new and challenging objects and experience *pleasure* in this play activity, as Freud (1955) argued, rather than be the subjects of uncanny, mildly traumatic experimental conditions and the spectators of adults' violence. Therefore, children seemed to compulsively repeat the activity in a ritualistic fashion. This view is in line with the emphasis given on *transformation* in Freud's (1946) definition of identification with the aggressor.

## Bandura's experiments on aggression in children, après-coup

The aggression modeling experiments were conducted at a time when Psychology was striving, by “objective” measurements and laboratory experiments, to establish itself as a discipline. They have received criticism because they certainly raise the ethical issue of children's exposure to violence, with unknown short- and long-term consequences. Ethical concerns have also been expressed for other groundbreaking, or even notorious, experiments in the history of Psychology (e.g., Watson's Baby Albert experiment, Milgram's experiments on obedience to authority).

Despite the ethical and methodological flaws, these aggression experiments and the short films that depict them continue to have a great allure to the scientific community and the society at large. Besides, a degree of seduction, namely *optimal seduction* (Potamianou, 2001), is needed to awaken desire for scientific exploration and facilitate openness to the unknown. They inspired research and interventions and raised public awareness about the effects of children's exposure to violence (e.g., through media). These experiments are still regarded to provide indisputable evidence, by means of a “rigorous experimental design”, for young children's vulnerability to adults' violence. They also illustrate that, from early on, humans are capable of abusive acts, and that these acts can be easily provoked. Therefore, the work of civilization is to undertake every action to protect children from the transmission of violence.

However, the fact that scientists' reservations were not strong enough to prevent them from “using” children in such laboratory experiments, implies, paradoxically, that they believed in children's resilience to violence or trauma. Only a few years after World War II, Psychology seemed to engage in an unconscious attempt at *reparation* (Klein, 1975), perhaps on behalf of the whole humanity, through handling—at last!—violence within a controlled and protected but regressed-to-the-infantile laboratory setting.

## Conclusions

This study aimed to approach Bandura's experiments on aggression modeling in children from the psychoanalytic perspective. A variety of psychoanalytic formulations were used to conceptualize the underlying processes and the phenomenology of children's imitation of aggressive acts. These formulations are not supported by research data, a fact that may be regarded also as a limitation of this study. However, they are based on the multitude and richness of clinical observations in the field of Psychoanalysis, which has an undeniably remarkable contribution to the understanding and treatment of human aggression.

## Author contributions

EG conceived the idea and drafted the manuscript. KM reviewed key findings of Bandura's experiments and systematically edited the manuscript. All authors contributed to the article and approved the submitted version.

## Conflict of interest

The authors declare that the research was conducted in the absence of any commercial or financial relationships that could be construed as a potential conflict of interest.

## Publisher's note

All claims expressed in this article are solely those of the authors and do not necessarily represent those of their affiliated organizations, or those of the publisher, the editors and the reviewers. Any product that may be evaluated in this article, or claim that may be made by its manufacturer, is not guaranteed or endorsed by the publisher.

## Footnotes

^1^ https://www.youtube.com/watch?v=eqNaLerMNOE

## References

[B1] Bandura A. (1965). Influence of model's reinforcement contingencies on the acquisition of imitative responses. J. Pers. Soc. Psychol. 1, 589–595. 10.1037/h0022070 14300234

[B2] Bandura A. (1969). “Social-learning theory of identificatory processes,” in Handbook of Socialization Theory and Research, ed D.A. Goslin (Chicago: Rand McNally), 213–262.

[B3] Bandura A. (1986). Social Foundations of Thought and Action: A Social Cognitive Theory. Englewood Cliffs, NJ: Prentice-Hall.

[B4] Bandura A. Huston A. C. (1961). Identification as a process of incidental learning. J. Abnorm. Soc. Psychol. 63, 311–318. 10.1037/h0040351 13864604

[B5] Bandura A. Ross D. Ross S. A. (1961). Transmission of aggression through imitation of aggressive models. J. Abnorm. Soc. Psychol. 63, 575–582. 10.1037/h0045925 13864605

[B6] Bandura A. Ross D. Ross S. A. (1963). Imitation of film-mediated aggressive models. J. Abnorm. Soc. Psychol. 66, 3–11. 10.1037/h0048687 13966304

[B7] Ferenczi S. (1949). Confusion of the tongues between the adults and the child (The language of tenderness and of passion). Int. J. Psychoanal. 30, 225–230. (Original work published 1933).33951841

[B8] Frankel J. (2002). Exploring Ferenczi's concept of identification with the aggressor: Its role in trauma, everyday life, and the therapeutic relationship. Psychoanal. Dialog. 12, 101–139. 10.1080/10481881209348657

[B9] Freud A. (1946). The Ego and the Mechanisms of Defence. New York, NY: International Universities Press. (Original work published 1936).

[B10] Freud S. (1953). “Three essays on the theory of sexuality,” in The Standard Edition of the Complete Psychological Works of Sigmund Freud, Vol. 7, ed Strachey J. (London: Hogarth Press), 125–243. (Original work published 1905).

[B11] Freud S. (1955). “Beyond the pleasure principle,” in The Standard Edition of the Complete Psychological Works of Sigmund Freud, Vol. 8, ed J. Strachey (London: Hogarth Press), 3–64. (Original work published 1920).

[B12] Howell E. F. (2014). Ferenczi's concept of identification with the aggressor: understanding dissociative structure with interacting victim and abuser self-states. Am. J. Psychoanal. 74, 48–59. 10.1057/ajp.2013.40 24603172

[B13] Klein M. (1975). “Love, guilt and reparation,” in Envy and Gratitude and Other Works 1921-1945. The Writings of Melanie Klein, Vol. I, ed M. Klein (New York, NY: Free Press) (Original article published 1937), 306–443.

[B14] Lacan J. (1977). The Four Fundamental Concepts of Psycho-analysis. London: Hogarth.

[B15] Laplanche J. (1999). Essays on Otherness. New York, NY: Routledge.

[B16] Potamianou A. (2001). Le Traumatique: Répétition et Élaboration. Paris: Dunod.

